# Effects of Shenqi compound on intestinal microbial metabolites in patients with type 2 diabetes: A protocol for systematic review and meta analysis

**DOI:** 10.1097/MD.0000000000023017

**Published:** 2020-11-25

**Authors:** Ran Xiong, Changying Zhao, Min Zhong, Xinxia Zhang, Wanfu Liu

**Affiliations:** a Hospital of Traditional Chinese Medicine Affiliated to Southwest Medical University; b Southwest Medical University, Luzhou; c Hospital of Chengdu University of Traditional Chinese Medicine; d Chengdu University of Traditional Chinese Medicine, Chengdu; e Yibin Hospital of Traditional Chinese Medicine, Yibin, Sichuan, China.

**Keywords:** meta analysis and systematic review, scheme, Shenqi compound, type 2 diabetes microcirculation disorder

## Abstract

**Introduction:**

According to the data from the 2017 Chinese Guidelines for the Prevention and Treatment of Type 2 Diabetes [Chin J Diabetes. 2017;20:81–117],^[1]^ in 2013, the incidence of T2DM in China was 10.4%, while nearly 63% of the patients did not receive standard diagnosis. T2DM has become a serious public health problem in China and even in the world. Intestinal flora, as a research hotspot related to T2DM and other diseases in recent years, is a kind of microorganism with a large number in the human intestinal tract, which is considered as one of the important factors affecting the metabolism of the endocrine system and the human internal environment. In fact, many concepts of traditional Chinese medicine (TCM) coincide with modern research results of intestinal flora. In fact, TCM is also widely used to regulate intestinal flora disorders, and plays a very important role in restoring the dysfunctional intestinal flora [Hu et al. Drug Eval. 2013:8–10].^[2]^ T2DM is a chronic systemic progressive disease. Studies [Wang et al. Tianjin Chin Med. 2007;24:507–508]^[3]^ have shown that even ideal blood glucose control cannot prevent the failure of islet cells [Wang et al. Tianjin Chin Med. 2007;24:507–508],^[3]^ and how to restore the function and number of islet cells has naturally become the focus and difficulty of our current research. Studies have shown that the changes in the contents of intestinal microflora and their metabolites are closely related to the performance of T2DM such as hyperglycemia, insulin resistance, and restoration of islet function, and play an important role in pathophysiological mechanisms such as chronic inflammation of T2DM [Sun et al. Shi Zhen Chin Med. 2012;23:1434–1436].^[4]^ It has been confirmed that Shenqi compound, a TCM, regulates intestinal flora of T2DM. However, due to the lack of evidence, there is no specific method or suggestion, it is necessary to make a systematic evaluation of Shenqi compound to provide effective evidence for further research.

**Methods and analysis:**

Electronic databases included PubMed, Embase, Cochrane Library, Web of Science, Nature, Science Online, WanFang China Biomedical Database, VIP Medical Information, China national Knowledge Infrastructure (CNKI).

Main results: Endotoxin, short-chain fatty acid, bile acid, indole.

Other results: low density lipoprotein (LDL), high density lipoprotein (HDL), triglyceride (TG), total serum cholesterol (TC). The data will be extracted independently by 2 researchers, and the risk of bias in the meta-analysis will be systematically evaluated according to the Cochrane handbook. All data analysis will be performed using the Data statistics software Review Manager V.5.3. And occupy V.12.0.

**Results:**

The results of this study will systematically evaluate the effectiveness and safety of Shenqi compound on the effects of intestinal flora metabolites in patients with type 2 diabetes.

**Conclusion:**

Through the systematic review of this study, the published evidence of the effect of Shenqi compound on intestinal flora metabolites in patients with type 2 diabetes was summarized to further guide its promotion and application.

**Ethics and communication:**

This study is a systematic review with findings based on published evidence and does not require erB review or consent. We plan to publish the results in a journal or conference report.

**Open science framework (OSF) registration number:**

September 29, 2020. osf.io/gb3m2.(https://osf.io/gb3m2).

## 1 Introduction

Studies have shown that intestinal flora metabolites of endotoxin, Short chain fatty acid (Short - chain fatty acids, SCFA), secondary bile acid, indole can regulate the secretion of glp-1 through participation and participate in regulating blood sugar, promote insulin hormone is postprandial intestinal digestive tract secretion of hormone, is one of the most important 2 glucose insulin dependent sex polypeptide (GIP) and glp-1. Glp-1 is a protein and peptide component secreted by L cells in the nerve tissue of the ileum and colon in the pathological tissue. Its main effective effect is the regulation of blood sugar related to the concentration of blood sugar, and it can also affect the reduction of gastrointestinal peristalsis and reduce people's feeling of eating.^[5]^ Intestinal flora, as a “virtual organ,” has its own DNA system compared with the physical organ of the human body. Meanwhile, the anatomical structure of the digestive tract communicating with the outside world determines its characteristic that it is more susceptible to the interference of the outside environment, leading to great variation in species and quantity and affecting human health. Moreover because of the intestinal flora species and large number, the number of direct identification of intestinal flora and the types of change is a complex but suffering, for the process, so the traditional Chinese medicine (TCM) and their interaction and participate in regulating blood sugar may be a preferable to study pathologic process, can make us more intuitive understanding of TCM metabolic process in the role of the intestinal flora. An in-depth study on the relationship between the regulation of intestinal microbial metabolites and T2DM by TCM can make intestinal flora an important indicator or an effective target for the prevention and treatment of T2DM, providing a new idea and idea for future improvement and research on how to treat T2DM.

Traditional Chinese basic medicine classifies T2DM into the scope of “relieving thirst.” According to Chinese medicine, the main treatment directions for T2DM patients with qi and Yin deficiency and blood stasis syndrome are clearing deficiency heat, Yin supplementation and body fluid supplementation.^[5]^ Therefore, the deficiency of qi and Yin, combined with spleen deficiency and blood stasis, is one of the basic pathogenesis of the occurrence and development of diabetes. Therefore, the basic treatment method of invigorating Qi and nourishing Yin and the basic treatment principle of invigorating the spleen and activating blood circulation are derived.

## 2 Methods

### 2.1 Study registration

The protocol is preregistered with the Open Science Framework (OSF). September 29, 2020. osf.io/gb3m2. (https://osf.io/gb3m2). The program will follow the declarative guidelines for preferred reporting items in the PRISMAP program, where changes will be reported in a comprehensive review if necessary.

### 2.2 Inclusion and exclusion criteria for study selection

#### 2.2.1 Inclusion criteria

The inclusion criteria were all randomized controlled trials (RCTs), and Shenqi compound Chinese herbal medicine was mainly used to regulate intestinal flora of type 2 diabetes. The test language includes Only Chinese or English.

#### 2.2.2 Exclusion criteria

The following will be excluded:

(1) Patients with severe cardiovascular and cerebrovascular nervous system diseases or patients with mental diseases;
(2) patients with uncontrolled blood pressure (blood pressure >150/90 mm Hg);
(3) Patients prone to hypoglycemia;
(4) Participated in the clinical trials of drugs with influence on this study;
(5) Patients with impaired liver and kidney function;
(6) Women who are breast-feeding or pregnant;
(7) Anemia: Male HGB < LL0 g /L, female HGB < 100 g/L;
(8) Patients with a history of diabetic ketosis within 6 months,
(9) Patients currently using GLP-1 receptor agonists or DPP-4 inhibitors.

Note: Exclusions for any of the above.

### 2.3 Types of participants

The types of subjects included patients diagnosed as type 2 diabetes and dialectical TCM syndrome of qi and Yin deficiency combined with spleen deficiency and blood stasis,^[6]^ regardless of their degree. All patients should be treated with Shenqi compound, or the combination of Shenqi compound and other conventional treatment methods. There is no sense of gender, race, or education.

### 2.4 Experimental interventions

TCM Shenqi compound intervention is the main treatment method. Drugs that clearly affect intestinal flora and its metabolites should not be used.

### 2.5 Control interventions

Intervention measures include: lifestyle and diet and exercise intervention, lifestyle diet and exercise intervention on the basis of taking Shenqi compound. Joint interventions are allowed as long as all groups in a randomized trial receive the same joint intervention.

### 2.6 Types of measurement results

#### 2.6.1 main results

1. Endotoxin
2. short-chain fatty acid
3. bile acid
4. indole.

#### 2.6.2 Additional outcomes

1. Weight, BMI,
2. Low density lipoprotein (LDL)
3. High-density lipoprotein (HDL)
4. Triglycerides (TG)Total serum cholesterol (TC).

## 3 The data source

### 3.1 Electronic search

We searched the following databases to identify eligible studies: PubMed, Embase, Cochrane Library, Web of Science, Nature, Science on Line, Wanfang China Biomedical Database, Viper Medical Information, and China national Knowledge Infrastructure (CNKI). Time range: The starting time is determined according to the first existing literature, and the deadline is September 2020.

### 3.2 Other search resources

**Figure 1:**
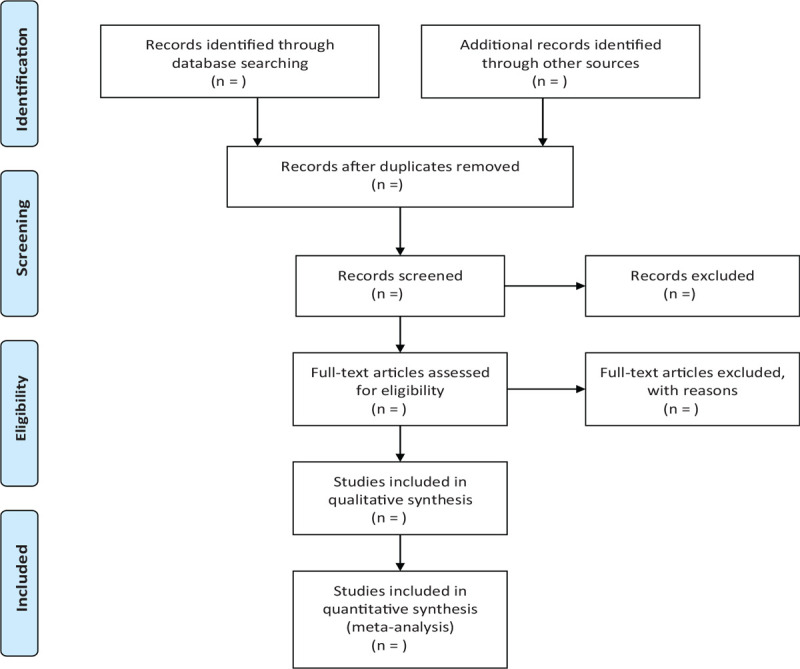
The research flowchart. This figure shows the identification, screening, eligibility and included when we searching articles.

In order to obtain more complete evidence, we will also manually search other relevant literature, such as medical textbooks and clinical laboratory manuals. If necessary, we will contact the trial authors to obtain the latest clinical data. In addition, studies relevant to the review will be identified through evaluation of relevant meeting minutes. The research flow chart is shown in Figure 1.

### 3.3 Search strategy

The following search terms will be used: randomized controlled trials/RCTs; Type 2 diabetes mellitus, type 2 diabetes mellitus intestinal flora, type 2 diabetes mellitus intestinal microorganisms; Metabolites of intestinal flora; Chinese Medicine/Chinese medicine; Ginseng astragalus compound. Different retrieval strategies are adopted in this paper. The language limit is Chinese and English. There are no publishing restrictions. Here we take the search strategy in PubMed as an example and list it in Table 1.

**Table 1:** Search strategy sample of PubMed.

Number	Searches
#1	Gastrointestinal Microbiomes (MeSh)
#2	Microbiome, Gastrointestinal (ti,ab)
#3	Gut Microbiome (ti,ab)
#4	Gut Microbiomes (ti,ab)
#5	Microbiome, Gut (ti,ab)
#6	or#1–5
#7	Shenqi compound prescription (MeSh)
#8	Shenqi compound prescription (ti,ab)
#9	Shenqi compound (ti,ab)
#10	TCM (ti,ab)
#11	or# 6–10
#12	Randomized Controlled Trial (MeSh)
#13	Randomized Controlled Trial (ti,ab)
#14	RCT (ti,ab)
#15	#12–14
#16	#6and #11and #15

## 4 Data collection and analysis

### 4.1 Research selection

All articles in the search results were independently evaluated by 2 independent researchers (RX, CYZ) based on inclusion criteria and exclusion criteria. The evaluator will then independently extract and collect the data included in the study using a predesigned data collection form. Differences will be discussed and resolved by consensus by the third author ().

### 4.2 Data extraction and management

The following information will be extracted from each study:

(1) Normal test features: title, author, year.
(2) Baseline data: sample size, age, gender, diagnostic criteria and course of disease.
(3) Intervention: Control and intervention of Shenqi compound preparations, details of intervention. If the information is insufficient, we will contact experts and authors in the field for relevant information.

### 4.3 Assess report quality and risk of bias

The bias risk was assessed by 2 independent authors (RX and CYZ), who completed the STRICTA checklist. The Cochrane Systematic Reviewers Handbook provides authors with criteria for evaluating the quality of RCTS. Assess the risk of bias:

(1) Random sequence generation;
(2) Allocate and hide;
(3) Blind participants and personnel;
(4) Blindness in result evaluation;
(5) Incomplete result data;
(6) Selective reporting of results;
(7) Other biases.

Any objections will be discussed or consulted by a third reviewer. Each will be described from 3 levels of “high risk,” “low risk” and “unclear.”

### 4.4 A measure of therapeutic effectiveness

Dichotomous results will be represented by odds ratios, while continuous data will be represented by standardized mean differences. All of these results report 95% confidence interval.

### 4.5 Missing data management

We will obtain the missing data by contacting the corresponding author. If there is no response, we will only analyze the existing data and describe the causes and effects of this exclusion in the paper.

### 4.6 Evaluation report deviation

Publication bias will be explored through funnel plot analysis. If the funnel graph is asymmetric, it will be assessed through Egger and Beg inspection, and *P* value <.05 indicates significant publication bias.

### 4.7 Evaluation of heterogeneity

There are mainly 2 methods to test heterogeneity, namely graphical method (funnel plot, forest plot) and statistical test (*Q* value statistical test, *I*^2^ statistical test, *H* statistical test). *I*^2^ statistical test showed that when *I*^2^ was 0, it indicated complete homogeneity of the study; if *I*^2^ was 50%, it indicated heterogeneity of the study.

### 4.8 Data synthesis and grading of evidence quality

The results will be analyzed using RevMan 5.0 software provided by the Cochrane Collaboration on Network. Binary data are represented by odds ratios and continuous data by mean difference. In order to test the heterogeneity of study results, when *I* < 50% or *P* > .1, the heterogeneity was significant. The random-effect model was used in the meta-analysis; otherwise, the fixed-effect model was selected.

### 4.9 Subgroup analysis

#### 4.9.1 Sensitivity analysis

Sensitivity analysis can not only assess the stability and reliability of the conclusions of meta-analysis, but also assess whether the changes in the results are related to the influence of a single study. If the stability of the conclusion is poor, we can realize that when the heterogeneity test result is heterogeneous, we need to clarify the source of heterogeneity through subgroup analysis. The effects of design plan, severity of disease, age, sex, mild and severe type 2 diabetes on intestinal flora regulation were analyzed. We will also remove studies of low and/or medium quality to check the robustness of the results. Improve stability by changing analysis models, inclusion and exclusion criteria, or by excluding a certain type of literature.

### 4.10 Morality and communication

We will publish systematic review results in peer-reviewed journals and disseminate them at conferences or in peer-reviewed publications. Aggregated published data will be used to cull personal data without the need for ethical approval or informed patient consent.

## 5 Discuss

Endotoxin (Leukapheresis products, LPS): LPS is part of the cell walls of gram-negative bacteria in the human body, usually in bad state of human body has a lot of LPS, the body of neutrophils, macrophages and endothelial cells can be stimulated by LPS and produce, these inflammatory substances produced a lot of times is not regulated, will lead to a series of related reactions, leading to human SIRS. Current studies have shown that LPS is an important substance of IR caused by intestinal bacteria, and the content of LPS in the blood of normal people is very low, while the content of LPS in the blood of T2DM patients increases significantly.^[7]^

Short chain fatty acids (Short chain fatty acid) volatile differ according to the carbon atoms are divided into acetic acid, propionic acid, metabolized by bacteria in the gut, is formed by the important products in the process of intestinal flora, can be quickly absorbed in the human gut, can give lots of energy, and intestinal cells in the dietary supplement Short chain fatty acids can improve blood sugar and insulin sensitivity, and insulin sensitivity and the effect of energy metabolism has been widely accepted, prove SCFA sugar to adjust to the human body, the other the readjustment of the regulation of the endocrine system has obvious.^[8,9]^ Short-chain fatty acid, a metabolite, has the function of enhancing small intestinal gluconeogenesis, inhibiting fasting induced adipogenic factors, and increasing LPL protein activity.^[10,11]^

Secondary bile acids: Secondary bile acids are derived from the metabolism of primary bile acids, which enter the intestines at the same time after the body ingests food. On the one hand, they are involved in the digestion and dissolution of lipids in food; on the other hand, they are converted into secondary bile acids in the biological metabolic reaction of bacteria in the digestive tract. The experiments of Sayin et al^[12]^ have shown that FXR can not only regulate the generation of grade bile acids, but also control the generation of node lipids, playing a very important role in transformation and metabolism. Both TGR5 and FXR are bile acid binding receptors, but TGR5 has a better binding degree with bile acid, and the binding of secondary bile acid to TGR5 can promote the production of GLP-1, thus achieving the effect of hypoglycemic treatment of T2DM.^[13]^

Indole: the mechanism of hypoglycemic effect to intestinal substance is its inhibition of K + channels and increase Ca2 + internal rheological change, thus increasing the secretion of glp-1. Amp activated protein kinase is a kind of heterologous trimer, it is a widely expressed serine threonine protein kinase, is composed of three subunits, including alpha belongs to the function of catalytic subunit, beta, and gamma subunit has a regulation, in regulating the balance of human body energy metabolism play a main switch function. In recent years, it has been found that^[13]^ insulin sensitization drugs mainly include metformin drugs (metformin) and thiazolidinedione, whose anti-diabetes effects are considered as new derivatives of indomethacin, which are realized through amp activated protein kinase signaling pathway.

Compared with single decoction, compound decoction, also known as Chinese traditional medicine compound, has a more significant regulatory effect on intestinal flora. In recent years, studies on intestinal flora metabolism have not only been limited to monomer components, but more importantly, effective transformation components of single and multiple Chinese traditional medicines are generated through intestinal flora metabolism. Such metabolites are usually difficult to be absorbed in the intestinal tract, but are susceptible to the transformation and metabolism of intestinal flora. Although their pharmacological activities are relatively small, their therapeutic effects can only be exerted after they are metabolized by intestinal flora, and we can call them “natural precursor drugs.”^[15]^ One of the characteristics of TCM in China is oral decoction, oral medicine in the human body will be in contact with intestinal flora. Many TCM really play the therapeutic effect is the glucoside components contained in it. We can directly see how TCM can achieve the hypoglycemic effect by regulating intestinal flora and improving the metabolites, providing a strong theoretical basis for the treatment of TCM in the future.

Intestinal flora can not only improve the therapeutic effect by metabolizing the effective components of TCM, but also have a toxic effect on compound toxic drugs. Wang Xiye^[16]^ analysis such as the use of radix aconiti respectively with the method of zhejiang fritillary, pinellia, Japanese ampelopsis after 3 ingredients such as compatibility, through under the bioconversion of rat intestinal bacteria, observing the variation in content of its effective pharmacological activities, results show that after compatibility, short chain fatty acids in the rat intestinal bacteria metabolites with system of double ester alkaloids of radix aconiti ester exchange reaction, through the exchange reaction can reduce the number of toxic metabolic substances double ester alkaloids, can also generate a contains therapeutic effect but little toxicity of ester type alkaloids. From these studies, we can see that the intestinal flora and its metabolites can make The Chinese medicine or compound play a significant role in reducing toxicity, and can also enhance the curative effect to some extent.

A large number of experimental results have proved that the therapeutic effect of compatible drugs on human body is completely different from that of single drugs through reasonable compatibility of Chinese medicines. It is not just the superposition effect of single Chinese medicines in our view, but more importantly, it shows that intestinal flora plays an important role in the metabolic transformation of Chinese medicines. From the direction of biological transformation, it can be explained that the process of metabolism of each single drug in TCM will also change through the action of intestinal bacteria in the biological body. And T2DM as modern medicine thinks diet related diseases, using TCM and the relationship of the intestinal flora, hypoglycemic effect of rising levels of metabolites, through the transformation of metabolic process of gut microbes can achieve modern chemical technology is difficult to achieve even unable to achieve changes in matter, makes the development of biotechnology and TCM research can reasonable application. At present, there are still many serious problems to be solved in the study of the metabolic mechanism of intestinal bacteria and effective components of TCM. In summary, systematic review and meta-analysis are helpful to determine the potential value of Shenqi Compound in the treatment of intestinal flora metabolites in patients with type 2 diabetes. This study can not only provide the basis for the release of diabetes treatment guidelines, but also promote the application of TCM prescriptions, so that more patients can benefit.^[14]^

## Author contributions

**Conceptualization:** Ran Xiong, Xinxia Zhang.

**Data curation:** Ran Xiong, Min Zhong, Wanfu Liu.

**Formal analysis:** Ran Xiong, Changying Zhao, Min Zhong.

**Methodology:** Ran Xiong, Min Zhong, Xinxia Zhang.

**Project administration:** Ran Xiong, Xinxia Zhang.

**Resources:** Ran Xiong, Min Zhong, Wanfu Liu.

**Software:** Ran Xiong, Changying Zhao.

**Supervision**: Iun Zhou.

**Writing – original draft:** Ran Xiong.

**Writing – review & editing:** Xinxia Zhang.
